# ROBIN: a randomised, double-masked, placebo-controlled Phase IIa study of the AOC3 inhibitor BI 1467335 in diabetic retinopathy

**DOI:** 10.1038/s41433-024-03017-0

**Published:** 2024-05-28

**Authors:** Quan Dong Nguyen, Justis P. Ehlers, David S. Boyer, Xidong Jin, Andrea Giani, Michael S. Ehrlich, On behalf of the ROBIN study investigators, Alexander Brucker, Allen Hu, Amani Fawzi, Andrew Antoszyk, Brian Berger, Chirag Jhaveri, Claire Bailey, David Brown, Derek Kunimoto, Faruque Ghanchi, Francesco Bandello, Geeta Menon, Harsha Sen, James Talks, João Figueira, Jose Juan Escobar Barranco, Juan Donate Lopez, Maged Habib, Maja Gran Erke, Martin Weger, Matthew Cunningham, Monica Varano, Nonavinakere Manjunatha, Paul Hahn, Pilar Calvo, Pravin Dugel, Raj Maturi, Richard Rosen, Rufino Silva, Sergio Pagliarini, Sobha Sivaprasad, Sofia Androudi, Sunil Patel

**Affiliations:** 1 Byers Eye Institute, Stanford University School of Medicine, Palo Alto, CA USA; 2 Cole Eye Institute at Cleveland Clinic, Cleveland, OH USA; 3 Retina-Vitreous Associates Medical Group, Los Angeles, CA USA; 4 Boehringer Ingelheim Pharmaceuticals, Inc., Ridgefield, CT USA; 5 Boehringer Ingelheim International GmbH, Ingelheim, Germany; 6 Alexion Pharmaceuticals, Inc., New Haven, CT USA; 7 Scheie Eye Institute, Philadelphia, PA USA; 8 Cumberland Valley Retina Consultants, Hagerstown, MD USA; 9 Northwestern Medical Group, Glenview, IL USA; 10 Charlotte Eye Ear Nose and Throat Associates, Charlotte, NC USA; 11 Retina Research Center, Abilene, TX USA; 12 Austin Research Center for Retina, Austin, TX USA; 13 Bristol Eye Hospital, Bristol, UK; 14 Retina Consultants of Texas, Houston, TX USA; 15 Retinal Consultants of Arizona, Phoenix, AZ USA; 16 Bradford Royal Infirmary, Bradford Teaching Hospitals NHS Foundation Trust, Bradford, UK; 17 La Fondazione Centro San Raffaele del Monte Tabor, Milan, Italy; 18 Frimley Park Hospital, Frimley Health NHS Foundation Trust, Frimley, UK; 19 Trinity Research Group, Dothan, AL USA; 20 Freeman Hospital, The Newcastle upon Tyne Hospitals NHS Foundation Trust, Newcastle upon Tyne, UK; 21 Espaço Médico de Coimbra, Coimbra, Portugal; 22 Hospital Dos de Maig, Barcelona, Spain; 23 Hospital Clínico San Carlos, Madrid, Spain; 24 Sunderland Eye Infirmary, City Hospitals Sunderland NHS Foundation Trust, Sunderland, UK; 25 Oslo University Hospital, Ullevål Hospital, Ullevål, Norway; 26 LKH University Hospital Graz, Graz, Austria; 27 Florida Retina Institute, Lady Lake, FL USA; 28 IRCCS Fondazione G.B. Bietti, Rome, Italy; 29 Hospital of St. Cross, University Hospitals of Coventry & Warwickshire NHS Trust, Rugby, UK; 30 Retina Associates of New Jersey P.A. and Retina-Vitreous Center, Bridgewater, NJ USA; 31 Miguel Servet University Hospital, Zaragoza, Spain; 32 Retinal Research Institute, Phoenix, AZ USA; 33 Midwest Eye Institute, Carmel, IN USA; 34 New York Eye and Ear Infirmary of Mount Sinai, New York, NY USA; 35 Moorfields Eye Hospital, Moorfields Eye Hospital NHS Foundation Trust, London, UK; 36 The European Interbalkan Medical Center, Thessaloniki, Greece; 37 Retina Research Institute of Texas, Abilene, TX USA; 38 Vall d’Hebron University Hospital, Barcelona, Spain; 39 CHLC - Hospital Santo António dos Capuchos, Lisbon, Portugal

**Keywords:** Diabetes complications, Eye diseases

## Abstract

**Objective:**

To evaluate the safety and efficacy of BI 1467335 in patients with non-proliferative diabetic retinopathy (NPDR).

**Methods:**

ROBIN is a Phase IIa, double-masked, randomised, placebo-controlled study (NCT03238963). Patients with NPDR and without centre-involved diabetic macular oedema were included; all had a best corrected visual acuity letter score of ≥70 Early Treatment Diabetic Retinopathy Study letters in the study eye at screening. Patients received oral BI 1467335 10 mg or placebo once daily for 12 weeks. Post-treatment follow-up was 12 weeks. The primary endpoint was the proportion of patients over the 24 weeks with ocular adverse events (AEs). Secondary endpoints were the proportion of patients with ≥2-step improvement from baseline in DRSS severity level at Week 12 and the proportion of patients with non-ocular AEs at 24 weeks.

**Results:**

Seventy-nine patients entered the study (BI 1467335, *n* = 40; placebo, *n* = 39). The proportion of patients with ocular AEs over 24 weeks was greater in the BI 1467335 versus the placebo group (35.0% vs 23.1%, respectively). Treatment-related AEs were reported for similar numbers of patients in the placebo and BI 1467335 group (7.7% vs 7.5%, respectively). At Week 12, 5.7% (*n* = 2) of patients in the BI 1467335 group had a 2-step improvement in DRSS severity level from baseline, compared with 0% in the placebo group.

**Conclusions:**

BI 1467335 was well tolerated by patients with NPDR. There was a high variability in DRSS levels for individual patients over time, with no clear efficacy signal.

## Introduction

Diabetic retinopathy (DR) is the most common microvascular complication of diabetes mellitus in the working-age population [1, 2]. Roughly one-third of all people with diabetes mellitus also have DR [3, 4]. Without treatment, DR can progress to severe non-proliferative DR (NPDR) or proliferative DR (PDR), resulting in a high risk for severe vision loss [5]. The DR severity scale (DRSS) assesses how DR worsens over time using stepwise severity categories (e.g., mild NPDR, moderate NPDR, moderately severe NPDR) and is based on fundus photography [6, 7].

The current gold-standard treatment for advanced DR, and in particular PDR, is a combination or individual use of panretinal photocoagulation and intravitreal anti-vascular endothelial growth factor (anti-VEGF) therapy [8, 9]. However, many patients do not respond fully to these treatments [10–14]. Furthermore, panretinal photocoagulation can be associated with loss of visual field, decreased night vision and increased macular oedema, and there is a burden associated with receiving intravitreal treatment that can lead to non-compliance and, in turn, lower efficacy [15, 16]. Gold-standard anti-VEGF therapies may also cause visual field loss, and therefore, there is interest in investigating treatments with less invasive modes of administration and other mechanisms of action compared with those currently available [17].

**Fig. 1:**
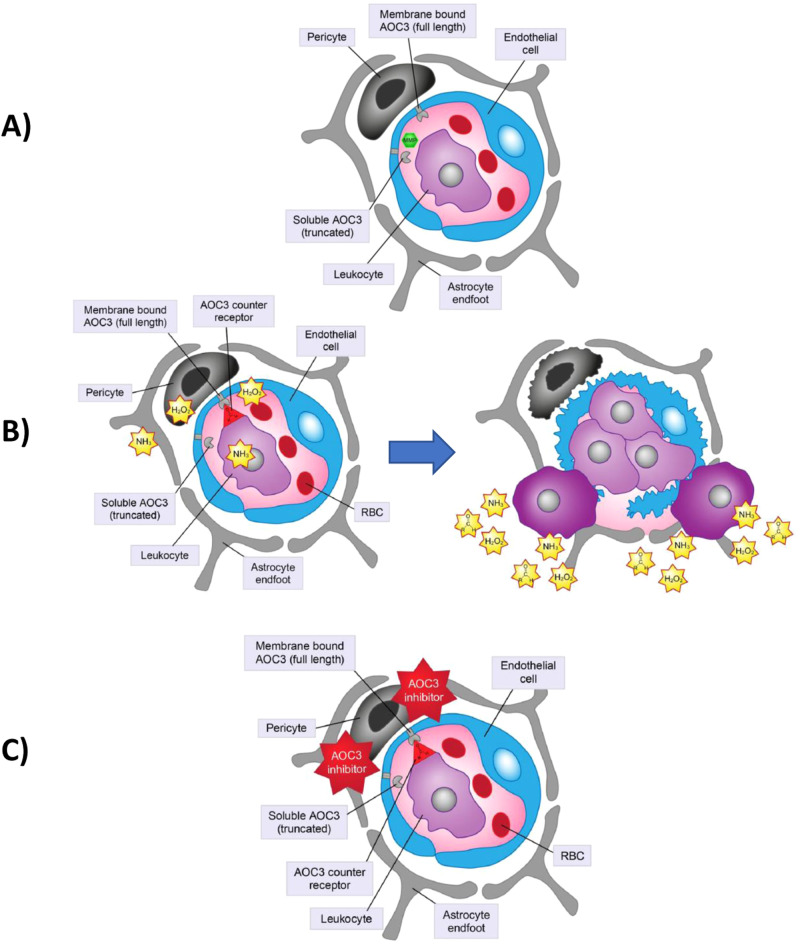
Amine oxidase copper-containing 3 (AOC3) mechanism of action and inhibition. **A** AOC3 rapidly translocates to the surface of endothelial cells during inflammation. **B** AOC3 on the retinal endothelium binds to the AOC3 counter-receptor on the leukocyte; aldehydes, ammonia and reactive oxygen species are released, which contribute to inflammation. **C** AOC3 inhibition reduces leukocyte recruitment, decreasing inflammation. *MMP* matrix metalloproteinase, *RBC* red blood cell. Figure adapted from Boyer DS, Rippmann JF, Ehrlich MS, Bakker RA, Chong V, Nguyen QD. Amine oxidase copper-containing 3 (AOC3) inhibition: a potential novel target for the management of diabetic retinopathy. Int J Retina Vitreous. 2021;7:30 [25].

DR is the result of chronic damage to retinal neurovascular structures and can lead to irreversible vision loss [1, 2]; changes in inflammation play a key role in the development of DR [18]. Secretion of proinflammatory cytokines (e.g., TNF-α 85, IL-1B, ICAM-1, angiotensin II, IL-6, VEGFs) is observed during the progression of DR [19, 20]. Proinflammatory cytokines enhance leukocyte adhesion to the endothelium; leukocytes themselves release inflammatory cytokines and vascular permeability factors, compromising the blood–retina barrier and leading to inflammation, ischaemia and oedema [21–24]. Amine oxidase copper-containing 3 (AOC3) is involved in leukocyte recruitment and translocates to the cell surface during inflammation, binding to leukocyte receptors and leading to the release of more proinflammatory molecules [21–23] (Fig. 1). Inhibition of AOC3 reduces leukocyte recruitment [21, 22]; therefore, AOC3 inhibition may improve or stabilise the retinal pathology of NPDR by correcting the underlying hypoxia, ischaemia and oedema [23, 25]. Furthermore, AOC3 levels are elevated in patients with diabetes mellitus and correlate with the presence of DR.

The ROBIN study was conducted to evaluate the safety and potential efficacy of BI 1467335, an oral AOC3 inhibitor, in the treatment of NPDR.

## Subjects and methods

### Study design and eligibility

This Phase IIa, double-masked, randomised, placebo-controlled study (NCT03238963) was conducted across 35 centres in Austria, Norway, Portugal, Spain, the United Kingdom and the United States. The trial was carried out in accordance with the principles of the Declaration of Helsinki, Independent Ethics Committees of participating centres, and the ICH GCP and with applicable regulatory requirements and Boehringer Ingelheim’s standard operating procedures. Prior to any trial-related procedure, all patients provided written informed consent. Patients, investigators and everyone involved in trial conduct or analysis were masked with regard to the randomised treatment assignments until after database lock. This study included patients with NPDR (DRSS level of 47 or 53) and without centre-involved diabetic macular oedema (DMO). As this was an exploratory study, no formal sample size calculation was applied. It was expected that using 50 patients per group would be sufficient to detect clinically relevant signals for ocular events between active treatment and placebo.

All included patients had a best corrected visual acuity (BCVA) Early Treatment Diabetic Retinopathy Study (ETDRS) letter score of ≥70 in the study eye at screening. DRSS was formally assessed at the Ocular Imaging Research and Reading Center (OIRRC; Sunnyvale, CA, USA). Patients with retinal, iris or angle neovascularisation of the study eye were excluded. Patients who had received prior treatment of DMO or DR with a macular laser within 3 months prior to screening, intraocular injections 6 months prior to screening or ≥4 prior intraocular injections in the study eye were also excluded. Eligible patients were randomised 1:1 to receive either BI 1467335 or placebo in a masked fashion via interactive response technology. Treatment arm randomisation was stratified by disease severity level (assessed by DRSS). In this study, DRSS levels were categorised stepwise by severity from 1 (DRSS 10) to 8 (DRSS 60), with 1 being ‘DR absent’ and 8 being ‘proliferative DR’ (Supplementary Table 1).

### Dosing

Patients received either 10 mg of oral BI 1467335 or placebo once daily for 12 weeks (2 × 5 mg film-coated tablets). Medication was administered on site at Day 1, Day 28 (Week 4) and Day 56 (Week 8) of the study. At home, trial medication was self-administered by the patients at approximately the same time each morning. Patients were requested to bring all remaining trial medication including empty package material with them when attending visits. The study period was defined as 12 weeks of treatment plus 12 weeks of residual effect period (24 weeks total). Administration of local standard-of-care treatment (such as intravitreal treatment, peribulbar injections, and laser or other surgical treatment) was allowed in cases of clinically significant worsening of the disease and was to be considered in the event of vision loss of ≥5 letters.

### Endpoints

The primary endpoint was the proportion of patients with ocular adverse events according to Common Terminology Criteria for Adverse Events (CTCAE) over the 24-week study period. The secondary endpoints were the proportion of patients with non-ocular adverse events over 24 weeks and the proportion of patients with ≥2-step improvement from baseline in DRSS severity level at Week 12, as formally assessed by a centralised reading centre (OIRRC). Further efficacy endpoints included the proportion of patients with ≥2-step improvement in DRSS severity level from baseline at Weeks 4 and 8, the proportion of patients with 1- or ≥3-step improvement in DRSS severity level at Weeks 4, 8 and 12, and change from baseline in BCVA and contrast sensitivity at Weeks 4, 8 and 12. Concentration and activity of AOC3 in plasma were determined throughout the trial, with key parameters including absolute AOC3 concentration, AOC3 concentration relative to baseline per time point, and percentage change in AOC3 activity relative to baseline. Baseline level was defined as the AOC3 value prior to first trial drug administration.

### Safety

In addition to ocular adverse events, the safety and tolerability of BI 1467335 were assessed based on adverse event reporting, vital signs, 12-lead electrocardiogram (ECG) and standard safety laboratory parameters.

### Statistical analyses

No confirmatory hypothesis testing was planned; this study was considered exploratory. For primary endpoint analyses, the frequency and percentage of patients reporting ocular adverse events were summarised by treatment, severity grade, primary system organ class and preferred term. All other endpoints were analysed or summarised descriptively.

## Results

**Fig. 2:**
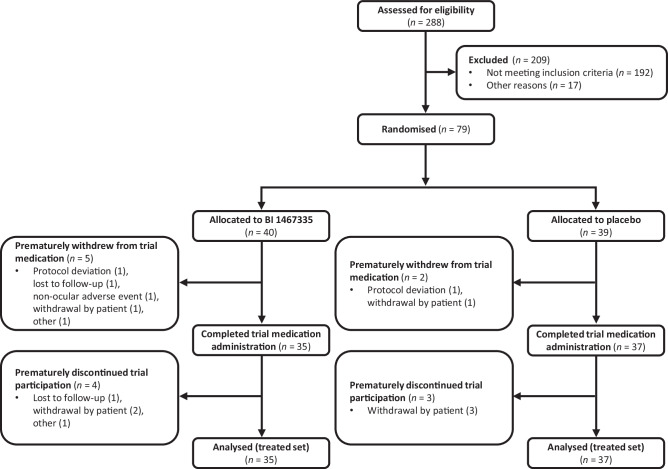
Patient disposition. A CONSORT flow diagram detailing the patient populations of the ROBIN study.

The first patient was enrolled into the trial on 2 November 2017, and the final patient completed follow-up on 14 May 2020. Of the 288 screened patients, 79 entered the study (BI 1467335, *n* = 40; placebo, *n* = 39) (Fig. 2). The majority of exclusions were the result of patients who failed to meet screening criteria at the time of DRSS grading. In total, 72 patients (BI 1467335, *n* = 35; placebo, *n* = 37) completed the trial medication, with seven prematurely discontinuing before 1 March 2020, coinciding with the COVID-19 pandemic. The treated analysis set comprised all patients who received at least one dose of trial medication (*n* = 79); the full analysis set comprised all patients in the treated analysis set with baseline data and at least one on-treatment measurement for BCVA or DRSS (*n* = 77); the per protocol set included all patients in the full analysis set without an important protocol deviation (*n* = 57). In addition, the pharmacokinetic set (*n* = 40) included all patients in the treated analysis set who had at least one relevant evaluable measure for BI 1467335 plasma concentration, and the ECG set (*n* = 79) included all patients with at least one baseline and one post-baseline ECG interval endpoint measurement. Mean treatment compliance was 98.7% (*n* = 79). Patients were well matched across all baseline characteristics between treatment arms, including BCVA and NPDR severity (Table 1).

**Table 1:** Baseline patient demographics.

Characteristic	BI 1467335 (*n* = 40)	Placebo (*n* = 39)	Total (*n* = 79)
Sex			
Female, *n* (%)	14 (35.0)	14 (35.9)	28 (35.4)
Male, *n* (%)	26 (65.0)	25 (64.1)	51 (64.6)
Age, years, mean (SD)	52.5 (10.8)	53.1 (13.3)	52.8 (12.1)
Race, *n* (%)			
White	36 (90.0)	31 (79.5)	67 (84.8)
Black/African American	3 (7.5)	3 (7.7)	6 (7.6)
Asian	1 (2.5)	4 (10.3)	5 (6.3)
American Indian/Alaskan native	0 (0.0)	1 (2.6)	1 (1.3)
BMI, kg/m^2^, mean (SD)	32.5 (6.5)	29.7 (4.9)	31.1 (5.9)
Duration of DR, years, mean (SD)	3.49 (6.19)	2.22 (3.57)	2.87 (5.09)
HbA1c, mean (SD)	8.49 (1.65)	8.50 (1.60)	8.50 (1.63)
BCVA study eye, number of letters, mean (SD)	81.5 (5.5)	82.2 (5.7)	81.8 (5.6)
BCVA fellow eye, number of letters, mean (SD)	77.6 (16.2)	79.3 (11.6)	78.4 (14.0)
DRSS classification (level) study eye, *n* (%)			
Mild NPDR (≤35)	0 (0.0)	1 (2.6)	1 (1.3)
Moderate NPDR (43)	7 (17.5)	7 (17.9)	14 (17.7)
Moderately severe NPDR (47)	14 (35.0)	15 (38.5)	29 (36.7)
Severe NPDR (53)	19 (47.5)	15 (38.5)	34 (43.0)
PDR (60)	0 (0.0)	1 (2.6)^a^	1 (1.3)

*BCVA* best corrected visual acuity, *BMI* body mass index, *DR* diabetic retinopathy, *DRSS* diabetic retinopathy severity scale, *NPDR* non-proliferative diabetic retinopathy, *PDR* proliferative diabetic retinopathy, *SD* standard deviation.

^a^Patient was at level 53 at the screening visit and at level 60 at the baseline visit.

### Safety

The proportion of patients with ocular events over 24 weeks was higher in the BI 1467335 group (35.0%) than in the placebo group (23.1%) (Table 2; Supplementary Table 2). One patient in the BI 1467335 group reported a serious ocular event (reduced visual acuity). No ocular events of CTCAE Grade >3 were reported, and the majority of ocular events were classified as Grade 1. Ocular events classified as drug related were reported in two patients, one per treatment arm (vitreous cells and blurred vision).

**Table 2:** Summary of reported adverse events.

AE, *n* (%)	BI 1467335 (*n* = 40)	Placebo (*n* = 39)	Total (*n* = 79)
Any AE	25 (62.5)	32 (82.1)	57 (72.2)
Investigator defined drug-related AE	3 (7.5)	3 (7.7)	6 (7.6)
Nervous system disorders	1 (2.5)	2 (5.1)	3 (3.8)
Investigations	1 (2.5)	2 (5.1)	3 (3.8)
Gastrointestinal disorders	2 (5.0)	0 (0.0)	2 (2.5)
Eye disorders	1 (2.5)	1 (2.6)	2 (2.5)
Vitreal cells	1 (2.5)	0 (0.0)	1 (1.3)
Blurred vision	0 (0.0)	1 (2.6)	1 (1.3)
Respiratory, thoracic and mediastinal disorders	1 (2.5)	0 (0.0)	1 (1.3)
Severe AE (CTCAE Grade 3 or 4)	4 (10.0)	6 (15.4)	10 (12.7)
Infection/infestation (unspecified)	-	2 (5.1)	2 (2.5)
Urinary tract infection	1 (2.5)	-	1 (1.3)
Pneumonia	-	1 (2.6)	1 (1.3)
Investigations (unspecified)	1 (2.5)	-	1 (1.3)
Upper abdominal pain	-	1 (2.6)	1 (1.3)
Gamma-glutamyl-transferase increase	-	1 (2.6)	1 (1.3)
Hypoglycaemia	1 (2.5)	-	1 (1.3)
Macular oedema	-	1 (2.6)	1 (1.3)
Vision blurred	1 (2.5)	-	1 (1.3)
AE leading to discontinuation of the trial drug	1 (2.5)	0 (0.0)	1 (1.3)
TEOE			
Any	14 (35.0)	9 (23.1)	23 (29.1)
Drug related	1 (2.5)	1 (2.6)	2 (2.5)
Leading to discontinuation	0 (0.0)	0 (0.0)	0 (0.0)
Severe (CTCAE Grade 3 or 4)	1 (2.5)	1 (2.6)	2 (2.5)^a^
Common TEOE (>1 patients)			
Diabetic retinopathy	3 (7.5)	2 (5.1)	4 (5.1)
Conjunctival disorder	1 (2.5)	1 (2.6)	2 (2.5)
Macular oedema	1 (2.5)	1 (2.6)	2 (2.5)
Retinal telangiectasia	1 (2.5)	1 (2.6)	2 (2.5)
Vision blurred	1 (2.5)^b^	1 (2.6)	2 (2.5)
Visual acuity reduced	2 (5.0)	0 (0.0)	2 (2.5)

Data indicates frequency of patients with AEs; patients may be counted in more than one category.

*AE* adverse event, *CTCAE* Common Terminology Criteria for Adverse Events, *TEOE* treatment-emergent ocular event.

^a^Macular oedema (placebo) and blurred vision (BI 1467335), both Grade 3.

^b^Grade 3.

Treatment-emergent non-ocular events were reported for a higher proportion of patients receiving placebo (82.1%) than those receiving BI 1467335 (55.0%). Serious non-ocular events occurred in 15.0% of patients receiving BI 1467335 and 10.3% of patients in the placebo group; one non-ocular event (acute cholecystitis) of a BI 1467335 patient, assessed as not being drug related, was considered to be Grade 4. The most frequent adverse events by preferred term were headache, nausea, nasopharyngitis, DR and cough. Seven patients (BI 1467335, *n* = 5; placebo, *n* = 2) stopped treatment early, none due to ocular events: two patients withdrew from the trial (BI 1467335, *n* = 1; placebo, *n* = 1), two patients deviated from the protocol (BI 1467335, *n* = 1; placebo, *n* = 1), one patient in the BI 1467335 group was lost to follow-up, one withdrew due to unspecified reasons, and one withdrew due to a non-ocular adverse event. No relevant changes in haematology, electrolytes, enzymes, substrates, plasma proteins or urine analysis were detected for either treatment group.

### Efficacy

**Fig. 3:**
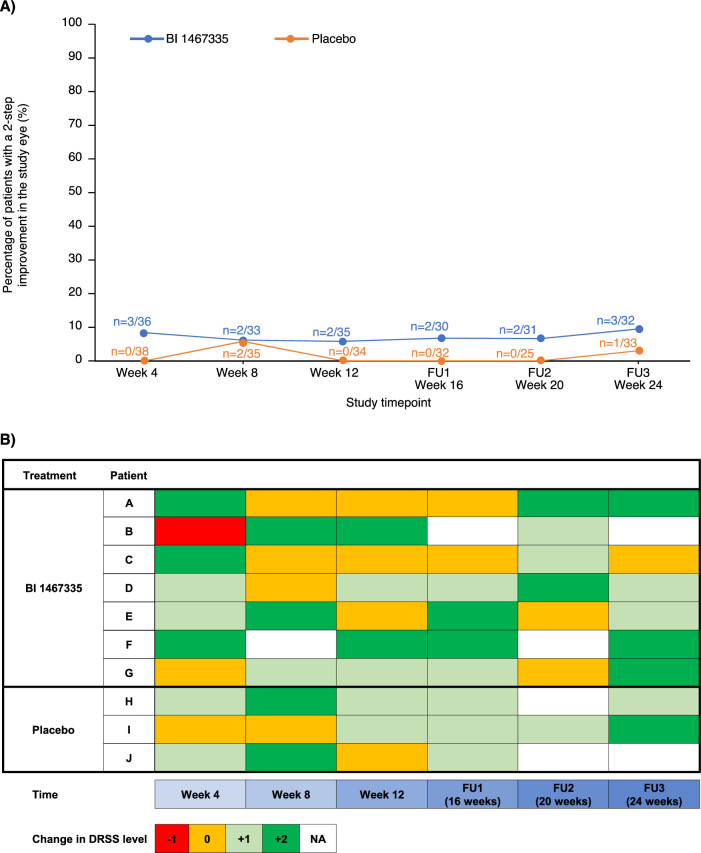
Changes in *DRSS* over time. **A** Proportion of patients in the full analysis set with 2-step improvement in *DRSS* severity level in the study eye over time (%). **B** Oscillation of *DRSS* from baseline measurement for individual patients who at any point in time showed 2-step improvement in *DRSS* severity level. *DRSS* diabetic retinopathy severity scale, FU follow-up, NA not available.

At Week 12, 5.7% of patients receiving BI 1467335 (*n* = 2/35) had 2-step improvement in DRSS severity level from baseline, relative to none in the placebo group; the risk difference (standard error) of BI 1467335 versus placebo was 0.057 (0.039). Over the course of the study, the percentage of BI 1467335-treated patients with 2-step improvement in DRSS severity level varied from 5.7% to 9.4%; for those receiving placebo it varied from 0% to 5.7% (Fig. 3A). At Week 12, most patients had either no change in DRSS (60.0%) or 1-step improvement (20.0%); 14.3% had 1-step worsening, and no improvement was larger than 2 steps. Oscillation of DRSS severity levels was observed for individual patients over the 24-week study period, with one patient ranging between 1-step worsening and 2-step improvement over the trial period (Fig. 3B).

No relevant changes in BCVA or contrast sensitivity were observed in any treatment group at any time point.

### Pharmacodynamic results

A maximum AOC3 inhibition (relative to baseline) of 95% was observed 2 h after the first 10 mg dose of BI 1467335 and was maintained up to Day 85 (median inhibition range: 86.5–99.8%; Supplementary Figs. 1 and 2), whereas placebo administration did not generally affect AOC3 activity. Over the on-treatment period, 86.5% to 99.8% inhibition of AOC3 activity was achieved. AOC3 activity returned to approximately 87% of baseline activity at 12 weeks after the last dose.

## Discussion

BI 1467335 was well tolerated in this clinical study. Furthermore, treatment with BI 1467335 achieved >85% AOC3 inhibition. However, although a small number of patients (5.7%) in the treatment group had 2-step improvement in DRSS severity level at Week 12, most had either no change or 1-step improvement at Week 12.

Previous research suggests that AOC3 inhibition is an appropriate mechanism of action for the treatment of DR. Inhibition of AOC3 reduces leukocyte recruitment and therefore reduces inflammation [21, 22], a key driver of DR [18]. Two other oral AOC3 inhibitors have been examined in DR and related conditions: ASP8232 and RTU-1096 [26, 27]. Over 12 weeks, ASP8232 reduced retina and ocular hyperpermeability in preclinical studies but did not show benefit over ranibizumab for the treatment of DMO. The study group speculated that these findings may have been due to the inclusion of patients with significant centre-involved DMO and that the study duration might not be sufficiently long for the full beneficial effects to be realised. Patients with centre-involved DMO were excluded from the ROBIN study; however, the treatment duration was still short at only 12 weeks [27]. Administration of RTU-1096 reduces upregulation of ICAM-1 (a leukocyte adhesion molecule) in mouse model retinas [21]. Subsequent Phase I trials in 2015 demonstrated a reduction in the serum AOC3 levels of healthy volunteers. However, to date there are no signs of further RTU-1096 development [26].

The lack of consistent improvement over time observed in this trial may be attributed to a number of factors. First, the treatment and follow-up periods (3 and 6 months, respectively) may not have been sufficiently long to achieve or observe a consistent effect. Second, the patient group included may not have been at the optimal stage for intervention with AOC3; speculatively, retinal damage may have been too advanced or too early to observe an improvement in DRSS. Furthermore, recent findings have suggested that venous beading may not respond to anti-VEGF treatment in patients with NPDR [28]. As venous beading differentiates severe from moderately severe NPDR in some patients [29], excluding patients with venous beading may have improved the efficacy signal in this study. Finally, the lack of efficacy may be simply attributed to the compound itself not being efficacious in this indication.

One limitation of the study included the large proportion of patients who did not meet the DRSS grading criteria. This may have been due to the restrictive DRSS requirements for inclusion in the study, and as such, these patients were excluded. In some cases, poor imaging technique, low image quality and a lack of familiarity with the DRSS system led to inaccurate grading; the quality of images is critical when using DRSS. Furthermore, in some cases, the fluorescein angiograms subsequent to initial grading led to a change in image grading to a level outside of the study inclusion criteria.

The DRSS is a commonly used method to assess the severity of DR [6, 7]. To our knowledge, the ROBIN study is one of the few studies to have reported individual DRSS levels over a short time period (24 weeks; one measure reported every 4 weeks) rather than a mean measurement. This short-term endpoint was selected with a view towards identifying an early efficacy signal using a well-established means of assessing DR. During this study, no consistent effect on DRSS severity level was observed in patients treated with BI 1467335, and therefore is unlikely to be of benefit in DR. We observed high variability of DRSS severity levels for specific patients over time. Such observed variability may be explained by poor image quality or the reporting of individual DRSS levels at specific time points, and so it may not be reliable in the short term. Further study of the DRSS as a short-term metric may be useful for future research. As the DRSS scale was developed to measure progression of DR rather than improvement, and is not validated in the presence of a treatment [30], it is also possible that the DRSS scale is not ideal for assessing improvements in DR during a clinical trial. Future studies could consider a number of strategies to overcome the unclear DRSS signal observed in this trial, such as a longer study duration, larger sample size and data-smoothing methods; for example, assessing an aggregate of 2-step changes achieved over a pre-specified number of visits (e.g., count as ‘positive’ if a 2-step change in DRSS is observed twice or more in the last three visits).

Further development of BI 1467335 in NPDR has been halted based on the lack of a clear efficacy signal and risk of dose-dependent drug interactions of the compound in NPDR patients identified in another Phase I study [31].

## Conclusions

BI 1467335 was well tolerated by patients with NPDR; however, there was no consistent effect on DRSS severity level. Most patients had either no change, 1-step worsening or 1-step improvement. Fluctuations in DRSS level within subjects were noted between 4-week visits. Given the totality of results, the decision was made to halt programme development. In view of the unmet need for an oral treatment in NPDR, and the potential advantages for both patients and physicians of an orally administered treatment, we continue to explore other novel treatment options.

## Summary

### What is known about this topic

- Diabetic retinopathy (DR) is the most common microvascular complication of diabetes mellitus in the working-age population. Without treatment, DR can progress to severe NPDR or proliferative DR, resulting in a high risk for severe vision loss.

### What this study adds

- The ROBIN study evaluated the safety and efficacy of BI 1467335, an oral AOC3 inhibitor, in patients with NPDR. BI 1467335 was well tolerated but had no clear efficacy signal.

### Supplementary information

Supplementary materials

## Data Availability

To ensure independent interpretation of clinical study results and enable authors to fulfil their role and obligations under the ICMJE criteria, Boehringer Ingelheim grants all external authors access to relevant clinical study data. In adherence with the Boehringer Ingelheim Policy on Transparency and Publication of Clinical Study Data, scientific and medical researchers can request access to clinical study data, typically, one year after the approval has been granted by major Regulatory Authorities or after termination of the development programme. Researchers should use the https://vivli.org/ link to request access to study data and visit https://www.mystudywindow.com/msw/datasharing for further information.
