# Supplementary material for: ROBIN: a randomised, double-masked, placebo-controlled Phase IIa study of the AOC3 inhibitor BI 1467335 in diabetic retinopathy
Source: Eye (Lond). 2024 May 28;38(10):1861–9. doi: 10.1038/s41433-024-03017-0 (PMC11226676; doi:10.1038/s41433-024-03017-0)

**ROBIN: a randomised, double-masked, placebo-controlled Phase IIa study of amine oxidase copper-containing 3 inhibitor BI 1467335 in diabetic retinopathy**Quan Dong Nguyen^1^*, Justis P. Ehlers^2^, David S. Boyer^3^, Xidong Jin^4^, Andrea Giani^5^, Michael S. Ehrlich^4,6^
On behalf of the ROBIN study investigators


**Supplementary Table 1.** Stepwise categorisation of DRSS levels by DR severity.

| Step | DRSS | DRSS severity |
| --- | --- | --- |
| 1 | 10 | DR absent |
| 2 | 15 | DR questionable |
| 3 | 20 | Microaneurysm only |
| 4 | 35 | Mild NPDR |
| 5 | 43 | Moderate NPDR |
| 6 | 47 | Moderately severe NPDR |
| 7 | 53 | Severe NPDR |
| 8 | 60 | PDR |

*DR* diabetic retinopathy, *DRSS* diabetic retinopathy severity score, *NPDR* non-proliferative diabetic retinopathy, *PDR* proliferative diabetic retinopathy.

**Supplementary Table 2.** Treatment-emergent ocular events.

| TEOE, *n* (%) | BI 1467335 (*n* = 40) | | | Placebo (*n* = 39) | | |
| --- | --- | --- | --- | --- | --- | --- |
| Grade* | **1** | **2** | **3** | **1** | **2** | **3** |
| Any | 12 (30) | 1 (2.5) | 1 (2.5) | 6 (15.4) | 2 (5.1) | 1 (2.6) |
| Eye disorders | 11 (27.5) | 1 (2.5) | 1 (2.5) | 6 (15.4) | 1 (2.6) | 1 (2.6) |
| Diabetic retinopathy | 3 (7.5) | 0 | 0 | 2 (5.1) | 0 | 0 |
| Diabetic retinal edema | 2 (5.0) | 0 | 0 | 0 | 0 | 0 |
| Blepharal pigmentation | 0 | 0 | 0 | 1 (2.6) | 0 | 0 |
| Blepharitis | 0 | 0 | 0 | 0 | 1 (2.6) | 0 |
| Chorioretinopathy | 0 | 0 | 0 | 1 (2.6) | 0 | 0 |
| Conjunctival bleb | 0 | 0 | 0 | 1 (2.6) | 0 | 0 |
| Conjunctival disorder | 1 (2.5) | 0 | 0 | 1 (2.6) | 0 | 0 |
| Dry eye | 0 | 0 | 0 | 1 (2.6) | 0 | 0 |
| Macular edema | 1 (2.5) | 0 | 0 | 1 (2.6) | 0 | 0 |
| Eye pain | 0 | 0 | 0 | 1 (2.6) | 0 | 0 |
| Flat anterior chamber of eye | 0 | 0 | 0 | 1 (2.6) | 0 | 0 |
| Lacrimation increased | 0 | 0 | 0 | 0 | 1 (2.6) | 0 |
| Macular edema | 0 | 0 | 0 | 0 | 0 | 1 (2.6) |
| Meibomian gland dysfunction | 0 | 0 | 0 | 1 (2.6) | 0 | 0 |
| Photophobia | 0 | 0 | 0 | 1 (2.6) | 0 | 0 |
| Retinal haemorrhage | 0 | 0 | 0 | 1 (2.6) | 0 | 0 |
| Retinal telangiectasia | 1 (2.5) | 0 | 0 | 1 (2.6) | 0 | 0 |
| Vision blurred | 1 (2.5) | 0 | 1 (2.5) | 1 (2.6) | 0 | 0 |
| Chorioretinal disorder | 1 (2.5) | 0 | 0 | 0 | 0 | 0 |
| Conjunctival pigmentation | 1 (2.5) | 0 | 0 | 0 | 0 | 0 |
| Eye disorder | 1 (2.5) | 0 | 0 | 0 | 0 | 0 |
| Eye pruritis | 1 (2.5) | 0 | 0 | 0 | 0 | 0 |
| Retinal fovea disorder | 1 (2.5) | 0 | 0 | 0 | 0 | 0 |
| Retinal ischemia | 1 (2.5) | 0 | 0 | 0 | 0 | 0 |
| Retinal neovascularisation | 1 (2.5) | 0 | 0 | 0 | 0 | 0 |
| Visual acuity reduced | 1 (2.5) | 1 (2.5) | 0 | 0 | 0 | 0 |
| Visual impairment | 1 (2.5) | 0 | 0 | 0 | 0 | 0 |
| Vitreal cells | 1 (2.5) | 0 | 0 | 0 | 0 | 0 |
| Infections and infestations | 1 (2.5) | 0 | 0 | 0 | 0 | 0 |
| Eye infection | 1 (2.5) | 0 | 0 | 0 | 0 | 0 |
| Skin and subcutaneous tissue disorders | 0 | 0 | 0 | 0 | 1 (2.6) | 0 |
| Photosensitivity reaction | 0 | 0 | 0 | 0 | 1 (2.6) | 0 |
| Injury/poisoning/procedural complication | 0 | 0 | 0 | 1 (2.6) | 0 | 0 |
| Ocular procedural complication | 0 | 0 | 0 | 1 (2.6) | 0 | 0 |

*No ocular events of Grade >3 were reported.
*TEOE* treatment-related ocular event.

**Supplementary Fig. 1** Individual mean % amine oxidase copper-containing 3 (AOC3) activity relative to baseline over the on-treatment period following once-daily administration of 10 mg BI 1467335 up to Day 85. The time point labelled 998 represents the last sample of that study day prior to the patient leaving the site.


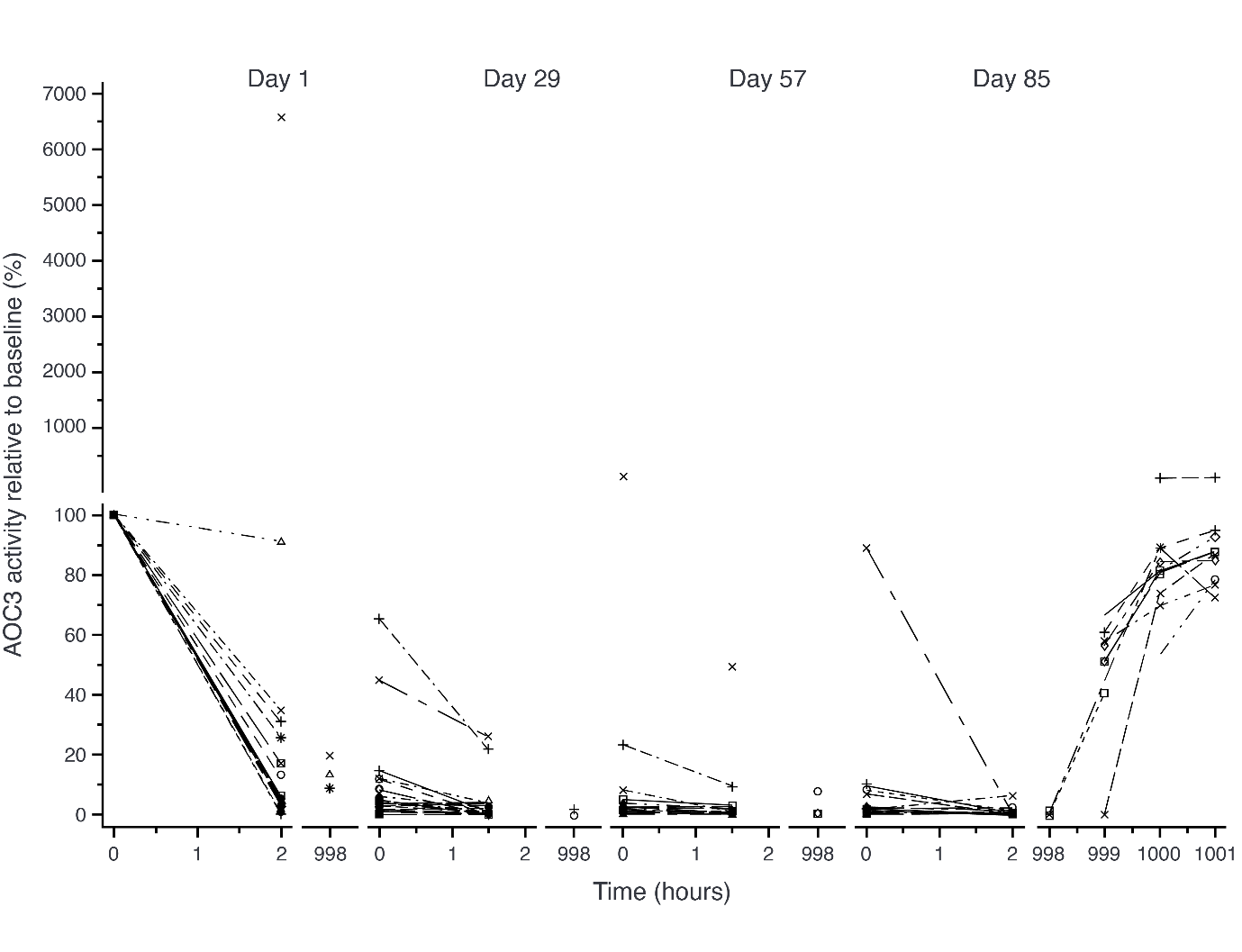


**Supplementary Fig. 2** Individual mean % amine oxidase copper-containing 3 (AOC3) activity relative
to baseline over the on-treatment period following once-daily placebo administration up to Day 85. The time point labelled 998 represents the last sample of that study day prior to the patient leaving the site.


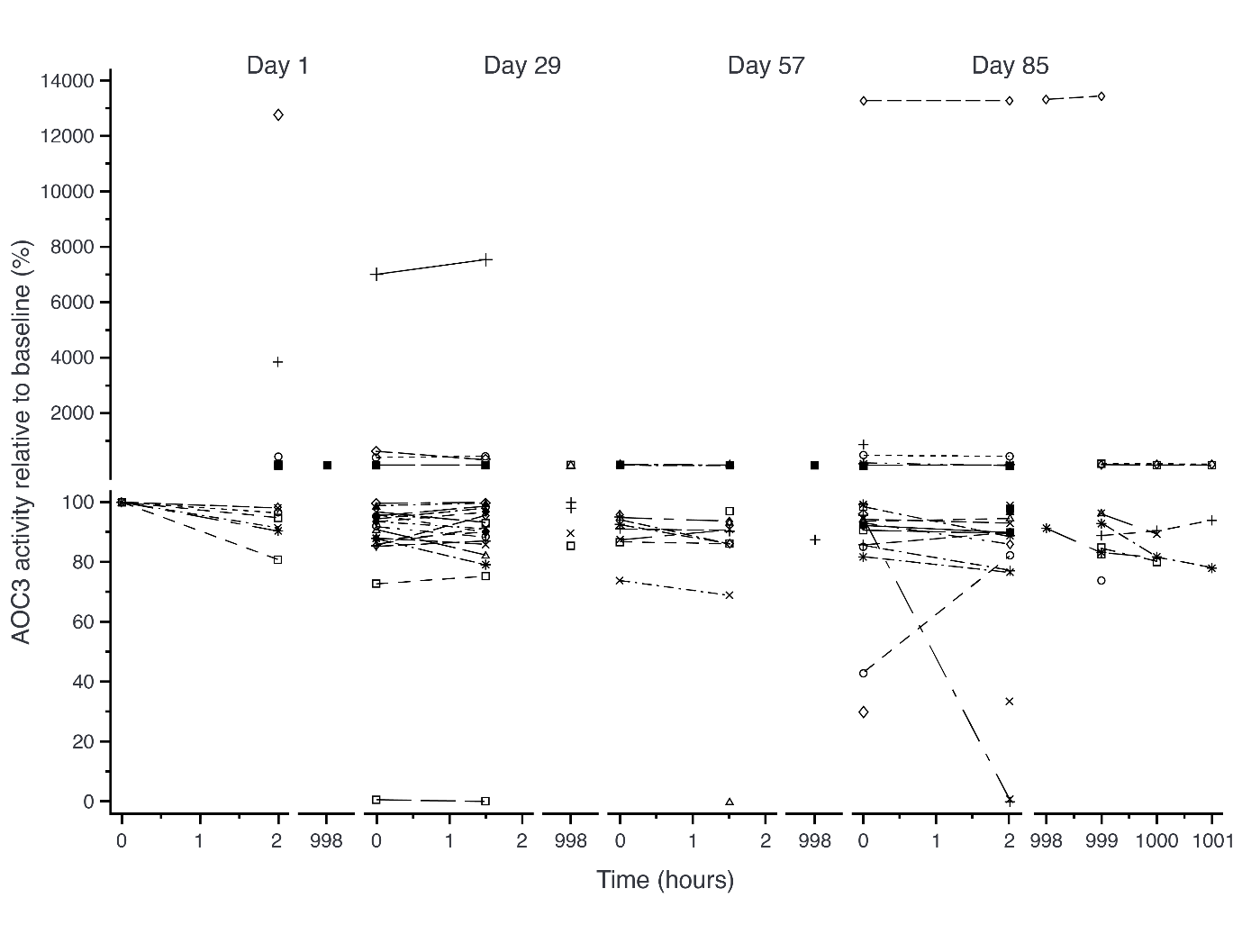

Supplement: Supplementary file 1 — Supplementary materials [file 41433_2024_3017_MOESM1_ESM.docx]
